# Long-Term Monitoring of Bioaerosols in an Environment without UV and Desiccation Stress, an Example from the Cave Postojnska Jama, Slovenia

**DOI:** 10.3390/microorganisms11030809

**Published:** 2023-03-22

**Authors:** Janez Mulec, Sara Skok, Rok Tomazin, Jasmina Letić, Tadej Pliberšek, Sanja Stopinšek, Saša Simčič

**Affiliations:** 1 Karst Research Institute, Research Centre of the Slovenian Academy of Sciences and Arts, Titov Trg 2, SI-6230 Postojna, Slovenia; 2 UNESCO Chair on Karst Education, University of Nova Gorica, Glavni Trg 8, SI-5271 Vipava, Slovenia; 3 Institute of Microbiology and Immunology, Faculty of Medicine, University of Ljubljana, Zaloška Cesta 4, SI-1000 Ljubljana, Slovenia; 4 Health Center Hrastnik, Novi Dom 11, SI-1430 Hrastnik, Slovenia

**Keywords:** aerobiology, biomass, beta-glucans, lipopolysaccharide, *Staphylococcus*

## Abstract

A natural cave environment subject to regular human visitation was selected for aerobiological study to minimize the effects of severe temperature fluctuations, UV radiation, and desiccation stress on the aerobiome. The longer sampling period of bioaerosols, up to 22 months, was generally not associated with a proportionally incremental and cumulative increase of microbial biomass. The culture-independent biomass indicator ATP enabled quick and reliable determination of the total microbial biomass. Total airborne microbial biomass was influenced by human visitation to the cave, as confirmed by significantly higher concentrations being observed along tourist footpaths (*p* < 0.05). Airborne beta-glucans (BG) and lipopolysaccharide (LPS) are present in cave air, but their impact on the cave remains to be evaluated. *Staphylococcus* spp., as an indicator of human presence, was detected at all sites studied. Their long-term survival decrease is likely due to high relative humidity, low temperature, the material to which they adhere, and potentially natural elevated radon concentration. The most commonly recorded species were: *S. saprophyticus*, which was identified in 52% of the studied sites, *S. equorum* in 29%, and *S. warneri* in 24% of the studied sites. Only a few isolates were assigned to Risk group 2: *S. aureus*,* S. epidermidis*,* S. haemolyticus*,* S. pasteuri*,** and *S. saprophyticus*.

## 1. Introduction

Natural air is a complex mixture of gases, vapours, inorganic and organic particles, metabolites, organisms, and their fragments [1,2,3]. It represents a rapidly changeable environment for aerobiome and provides a vehicle for its transmission [4]. This issue became particularly urgent during the 2020–2021 pandemic caused by the SARS-CoV-2 coronavirus. Airborne microorganisms are subject to regulatory monitoring in some environments and industries [5]. In hospitals, for example, routine microbiological surveillance is an essential part of the infection control programme recommended, especially for operating theatres and negative pressure rooms, to prevent healthcare-associated infections [6]. Unfortunately, there are no international guidelines or recommendations that prescribe the frequency or methodology of air sampling in the hospital environment, but there are regional and national recommendations on this topic, in addition to those presented in professional and scientific literature.

In many respects, the effects of bioaerosols on human health are still poorly understood [3]. As well as airborne microorganisms, the effects of exposure to airborne endotoxins and to beta-glucans are also significant health concerns [7]. Beta-glucans (BG) are natural polysaccharides composed of D-glucose monomers found in the cell wall of fungi, some bacteria, and plants. BG from fungi consists of a (1,3)-beta-linked D-glucose polymer with (1,6)-beta-linked side chains. It has immunomodulatory properties and can affect respiratory health if inhaled [8,9,10]. BG can be used as a marker of mould biomass in field studies [11,12]. Lipopolysaccharide (LPS) is a major component of the outer membrane of Gram-negative bacteria and consists of lipid A, core oligosaccharide, and O-polysaccharide [13]. LPS is one of the main components of organic dust, and its inhalation can cause various levels of pulmonary distress [9,10,14].

The aerobiome is exposed to natural stressors in the air, especially temperature fluctuations, UV radiation, and desiccation [15]. The question under investigation is how the absence of these stressors under natural conditions would affect the ecology of the aerobiome in the long term, in terms of its viability, dynamics, and interactions. Caves offer a natural environment that provides highly stable climatic conditions for the study of bioaerosols, because they are generally characterized by high humidity, stable temperature, and the absence of UV radiation [16]. Therefore, they are a near-ideal natural laboratory for carrying out aerobiological research. In addition, tourist caves can provide ideal sites for monitoring the fate of the aerosolized human-derived microbiota, its transmission, its longevity, and its response to long-term exposure to such conditions [17,18].

The current study presents the spatial quantification of airborne microbial biomass in a cave impacted by human visitation, with the aim of determining the most indicative parameter for use in microbial biomass monitoring. In addition to culturing, airborne biomass was estimated using ATP, BG, and LPS. Monitoring of airborne staphylococci gave information on their diversity and viability in relation to human presence. Because they are part of the human skin and mucosal microbiome, they are an excellent indicator of the level of human presence impact [19,20,21]. Previous studies have confirmed that their occurrence is associated with human cave visitation [22,23] and with the presence of bats [24].

## 2. Materials and Methods

### 2.1. Description of the Study Site

Postojnski jamski sistem (Postojna Cave System, 45°46′57″ N, 14°12′13″ E, 529 m a.s.l., total length 24 km) comprises several caves (Postojnska jama, Otoška jama, Magdalena jama, Pivka jama, Črna jama) and occupies at least 3,066,517 m^3^ of underground space, with some 1,231,716 m^2^ of potential contact surfaces [25]. A 5.0 km-long section of the dry part of Postojnska jama (Postojna Cave) is set up for tourist visits and includes a 3.2 km round trip on an underground railway. The Pivka River sinks into Postojnska jama, which is impacted by human activities from the surrounding area, as evidenced by localized elevated concentrations of sulphates, chlorides, and organic and faecal pollutants [26]. Tourist use of Postojnska jama is reflected in changes in the cave climate, crushed sand and metal dust deriving from the train wheels, surface pollution of tourist footpaths, ultrasonic smog from electrical equipment, and light eutrophication, with lampenflora growing around lamps [27,28,29,30,31,32]. To restore natural conditions in the cave and minimise human impact, some remedial measures were undertaken by the cave management, including regular removal of lampenflora (i.e., the microbial mat around the lamps) with a hydrogen peroxide solution, cleaning of walking surfaces with a water jet, and installation of a disinfection barrier at the cave entrance [27,30]. In the years before the SARS-CoV-2 epidemic, Postojnska jama had more than 700,000 visitors annually. Due to the pandemics that occurred in 2020–2021, visitation to the cave decreased dramatically; the cave was closed to tourists from 13 March 2020 to 3 June 2020 (82 days) and from 26 October 2020 to 3 June 2021 (220 days).

**Figure 1:**
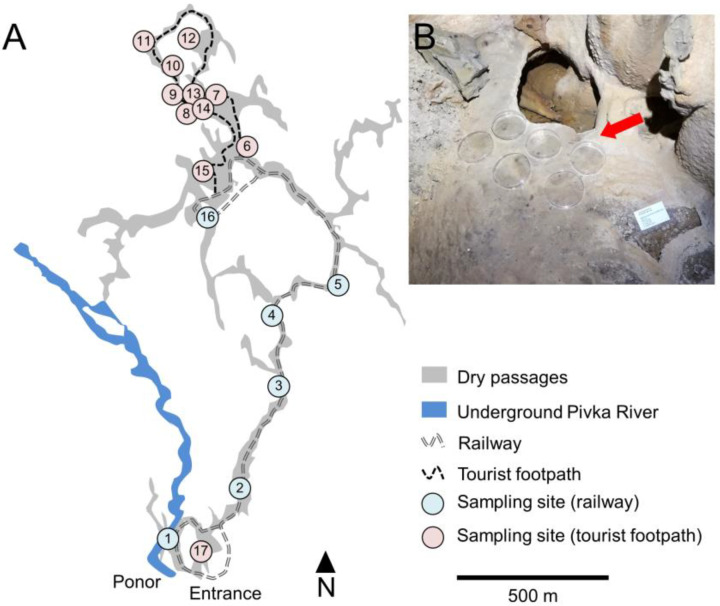
Setup of the experiment: (**A**)—a map of Postojnska jama with designated locations for bioaerosol sampling along the underground railway (1–5, 16) and tourist footpath (2–15, 17) (ground plan modified from the Cave cadastre of the Karst Research Institute ZRC SAZU); (**B**)—an example of open Petri plates for bioaerosol settling.

Seventeen sites were selected in the tourist part of the cave, six along the railway and eleven by the tourist footpath, where open, sterile, Petri plates (90 mm diameter) were exposed to the cave atmosphere (Figure 1). The Petri plates were closed during the periodic treatment to remove lampenflora. Three rounds of gravity-settling aerosol experiments were conducted in the cave. The first began on 23 January 2018; subsequently one Petri plate was collected from each site after three months (17 April 2018), six months (31 July 2018), and twelve months (24 January 2019). The second round began on 24 January 2019, and one plate was collected after seven months (19 August 2019), thirteen months (6 February 2020), and twenty-two months (16 November 2020). The third round (for airborne biomass variability testing only, with four plates per site) began on 2 April 2020 and lasted until 16 November 2020 (seven months).

### 2.2. Preparation of Samples

After the Petri plates were collected in the cave, they were sealed with parafilm and transferred to a laboratory. In the laboratory, 2 mL of saline solution was applied to each Petri plate, and the entire surface and rim were swabbed with a flocked swab (FLOQSwabs, Copan, Italy). Each swab was centrifuged at 4000 RPM for 10 min to release the associated liquid and biological particles. After centrifuging, the liquid was mixed with the remaining 2 mL of saline solution. Some of the Petri plates collected dripwater during exposure in the cave. In this case, the water from a Petri plate was collected in a separate tube and centrifuged at 13,000 RPM for 3 min. Solid particles concentrated as a pellet at the bottom of the centrifuge tube were resuspended in 20 µL of saline solution. Then 2 mL of the same cave water was applied to the just-emptied Petri plate, the surfaces swabbed, and the swab processed as described above. Finally, the liquid obtained was combined with the liquid in which the pellet had been resuspended. Each sample obtained in this way was then subdivided for cultivation and determination of ATP, (1,3)-β-D-glucan (BG), and lipopolysaccharide (LPS).

### 2.3. Estimation of Microbial Biomass and Air Velocity

ATP content in liquid samples was estimated using AquaSnap Total with a Hygiena Luminometer, and results were expressed in RLU—Relative Light Units (where 1 RLU corresponds to 1 fmol of ATP). During the 7-month experiment on the variability of settled airborne biomass, surfaces of Petri plates were swabbed directly with the UltraSnap (Hygiena, USA) and results expressed in RLU.

Samples were analysed using the modified Fungitell assay (Assay for BG in serum: Associates of Cape Cod Inc., USA) to determine the concentration of BG. They were analysed in duplicate without application of alkaline pretreatment solution, and results were expressed as mean values (pg/mL) and recalculated per surface (ng/20 cm^2^). If a categorical discrepancy was observed between parallel tests, with a standard deviation of 20 pg/mL or more, samples were retested.

The Limulus Amebocyte Lysate assay (LAL: Associates of Cape Cod Inc., USA) was used to quantify Gram-negative bacterial endotoxins (LPS). Control standard endotoxin was used to prepare dilutions of standard endotoxin from which the standard curve was plotted. Samples were tested in duplicate, and results were reported as mean value of Endotoxin Units per millilitre (EU/mL) and recalculated per surface (EU/20 cm^2^).

**Figure 2:**
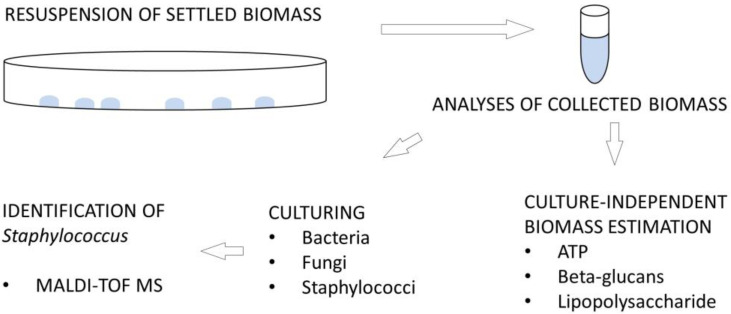
Schematic representation of the analyses performed on the samples.

Samples prepared at appropriate dilutions were plated onto three different media to propagate microbial colonies: nutrient agar (NA: Sigma, Taufkirchen, Germany) for bacteria, malt extract agar (MEA: Sigma, Germany) for fungi, and *Staphylococcus* chromogenic agar (STA: Conda Pronadisa, Spain) for *Staphylococcus* spp. NA and MEA plates were incubated aerobically at 20 °C for 7 days, and STA plates at 37 °C for 2 days. Visible colonies were quantified as Colony Forming Units (CFU) and calculated per surface (CFU/20 cm^2^). Colonies that exhibited colour typical of *Staphylococcus* were further purified and identified by MALDI-TOF MS (Matrix-Assisted Laser Desorption/Ionisation Time-Of-Flight Mass Spectrometry). Figure 2 provides an overview of the analyses performed on the samples.

Air velocity/flow was measured using a VelociCalc Air Velocity Meter 9535 (TSI Inc., Shoreview, MN, USA) from June 2020 to June 2021 (12 June 2020, 16 July 2020, 20 August 2020, 28 September 2020, 4 November 2011, 11 December 2020, 14 January 2021, 22 February 2021, 31 March 2021, 10 May 2021, 18 June 2021) to estimate which site is most exposed to natural airflow. The instrument measures temperature and velocity and gives the volumetric flow and actual/standard velocity (0 to 30 m/s).

### 2.4. Identification of Staphylococcus

Pure isolates from STA plates were subsequently inoculated onto 5.0% defibrinated sheep-blood agar (BA) and incubated at 36 ± 1 °C for 24 to 48 h. BA was prepared at the Institute of Microbiology and Immunology, Faculty of Medicine, University of Ljubljana, and contained 15 g agar (Sigma, Taufkirchen, Germany), 3.5 g brain-heart infusion broth (Becton Dickinson, Sparks, NV, USA), and 50 mL defibrinated reoxygenated sheep blood (Bio Gnost, Zagreb, Croatia) per 1000 mL. Bacterial isolates on the BA were identified using MALDI-TOF MS with an on-target formic acid extraction technique as previously described [30]. Biotyper RTC version 4.1 software was used for automated analysis of mass spectra. The quality of identification was assessed using the manufacturer’s score value (Bruker Daltonik, Billerica, MA, USA). A score of ≥2.000 indicated reliable identification at the species level, a score of 1.700 to 1.999 indicated reliable identification at the genus level, and a score of <1.700 was interpreted as unreliable identification. Identified *Staphylococcus* isolates were preserved at the Karst Research Institute ZRC SAZU. Microbial risk groups were assessed according to the Risk Group Database of the American Biological Safety Association.

### 2.5. Statistical Analyses

Statistical analyses were performed using IBM^®^ SPSS^®^ for Windows version 26 (SPSS Inc., IBM Company, Armonk, NY, USA), and Daniel’s XL Toolbox, an open-source add-in for Microsoft Excel (Version 6.60). Results were reported as mean ± standard deviation (SD). Statistically significant differences in concentrations of microbial indicators were estimated by the Independent-Samples *t*-test. *p*-values less than 0.05 were considered statistically significant.

For gravity-settling aerosol experiments (January 2018–January 2019, January 2019–November 2020), the trend of settling biomass (cultivable bacteria, fungi, *Staphylococcus*, and concentration of ATP, BG, and LPS) was evaluated as follows. When the biomass parameter increased throughout the experimental period, it was referred to as biomass accumulation (+). When the biomass concentration decreased, it was referred to as biomass reduction (−), and when the trend could not be determined, the result was referred to as equivocal (±).

## 3. Results

### 3.1. Bioaerosols in the Cave Air

A closer look at one-year measurements of air temperature and velocity revealed dynamic conditions with high variability at some locations (Table S1 in Supplementary Materials). Airflow was higher in the main passage where a tourist train operates, corresponding with lower average temperatures (<10.0 °C). The exception in this part is the site closest to the cave entrance (No. 1), with the Pivka River having a significant impact at this micro-location. Climatic conditions were also not constant in the tourist walking area. Lower average air temperatures (<10.0 °C) at some sites (nos 12, 13, 14) with locally notable air flow were attributed to the complex cave morphology, i.e., ramification of passages on two levels and interconnected large chambers (Figure 1).

**Figure 3:**
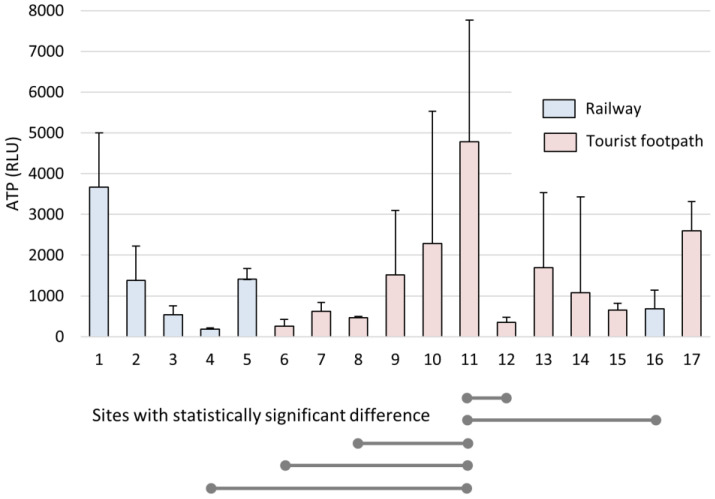
Airborne biomass expressed in ATP during the 7-month settling experiment per individual sites (1–17). The results are presented as the mean ATP (RLU) with SD. The lines indicate which two places were compared and showed statistically significant differences (*p* < 0.05) in ATP concentrations.

A 7-month bioaerosol settling experiment was designed to measure the quantitative variability of settled aerosol particles at each site. High concentrations of settled microbial biomass were found at some sites along the tourist walking areas (nos 9, 10, 11, 13, 14, 17), along the river (No. 1), and at two sites along the tourist railway (nos 2, 5). It is worth noting that the cave was closed to visitors for 83 days during the 228-day experiment (from 2 April to 3 June 2020, and from 26 October to 16 November 2020), so comparable elevated concentrations at some sites cannot simply be associated with the presence of tourists and the train, but also with natural air circulation and sedimentation of bioaerosols. The coefficient of variation (CV) of ATP ranged from 8.0% to 218.9% but, interestingly, the highest coefficients of variation were along the tourist footpath (nos 9, 10, 13, 14). Along the railway, the variability was significantly lower, indicating a relatively uniform settling of bioaerosols (37.3%—mean ATP CV% of all sites along the railway; 70%—mean ATP CV% of all sites along the tourist pathway, *p* = 0.036) (Figure 3, Table S1 in Supplementary Materials).

Two series of bioaerosol settling experiments were conducted in the cave. The second (2019–2020) was designed to confirm the settling trends indicated by the first round (2018–2019), because a longer collection period was generally not associated with higher levels of captured biomass. Accumulation of settled bioaerosols in the form of bacteria, fungi and LPS with prolonged exposure of Petri plates was observed at only about one-third of the experimental sites. The trend of accumulation was observed at most of the experimental sites for BG (52.9%). In most of the experiments, there was no clear trend (Table S2 in Supplementary Materials). A tendency for *Staphylococcus* biomass to accumulate was observed at only 5.9% of the experimental sites.

The significantly higher concentrations of settled microbial indicators (ATP, fungi, bacteria, LPS and BG; all *p* < 0.05) were along the tourist footpath. Site No. 10 was particularly rich in microbial biomass with the highest concentration of ATP (7318 RLU, 2019–2020: 13 months), bacteria (13,518,346 CFU/20 cm^2^, 2019–2020: 13 months), and *Staphylococcus* spp. (1462 CFU/20 cm^2^, 2019–2020: 7 months). The highest concentration of fungi was 420,012 CFU/20 cm^2^ and BG 4,729,535 pg/20 cm^2^, both at site No. 13 (2018–2019: 12 months), i.e., the lowest point of the tourist route in Postojnska jama. At this site, all subcellular indicators showed the trend of accumulation (Table S2 in Supplementary Materials). The highest LPS was 608,376 EU/20 cm^2^ at site no. 8 (2018–2019: 6 months) (Table S3 in Supplementary Materials, Figure 4).

A Pearson correlation analysis was performed between the biomass estimators. As expected, there was a very strong positive and statistically significant correlation between BG and fungal count (r = 0.863), because BG makes up a large portion of fungal cell walls [33]. There was also a strong positive correlation between fungal and bacterial concentrations (r = 0.588). ATP concentration correlated with all other biomass estimators and would, therefore, be the best choice for estimating bioaerosol concentration. LPS showed only a weak positive correlation with ATP because it relates only to Gram-negative bacteria (Table 1).

### 3.2. Staphylococcus as an Indicator of the Human Microbiome in Cave Air

*Staphylococcus* spp. were detected in the settled aerosols at all sites in the cave (Table S2 in Supplementary Materials). Their concentrations were generally higher along the tourist walking areas than along the railway (10.6 ± 31.8 CFU/20 cm^2^ and 41.8 ± 187.1 CFU/20 cm^2^, respectively). It appears that *Staphylococcus* viability decreased over several months. Identified isolates from cave aerosols are frequently associated with the human microbiome as commensals and opportunistic pathogens. During the current study, the most commonly encountered staphylococci were: *S. saprophyticus*, which was identified at 9 of 17 sites (52%), *S. equorum* at 5 of 17 sites (29%), and *S. warneri* at 4 of 17 sites (24%) (Table 2).

## 4. Discussion

### 4.1. Characteristics and Dynamics of Settled Bioaerosols

Cave ventilation has a significant influence on the spatial distribution of aerosols [34,35]. The dynamic conditions of the cave climate, especially between winter and summer, in Postojnska jama have already been demonstrated using airborne microorganisms as natural tracers [15]. Biomass accumulation was expected during long-term experiments, despite seasonal variations in bioaerosol concentrations [15]. For example, amounts of airborne fungi in caves are known to exhibit pronounced seasonal variations, with higher concentrations in the warmer seasons [36,37]. Visiting caves is associated with an increase in airborne microbes [17]. In a previous study of a 34-day bioaerosol deposition experiment in Postojnska jama, the highest ATP concentration was observed along the tourist footpath [25]. Postojnska jama is not a typical “self-purifying” cave, where fungal concentration gradually decreases from the entrance to the interior of the cave [38].

Despite the absence of UV and desiccation stress, microbial viability declines over the longer term (>3 months) if the microbes do not find a suitable environment or host to proliferate. However, natural radiation, especially radon and its decay products, is another important environmental parameter that affects microorganisms and their metabolism [39]. Radon concentrations in most karst caves are significantly higher than those encountered across most of the earth’s above-ground ventilated surfaces. In Postojnska jama, for example, there is clear link between radon concentration and the efficiency of natural or imposed cave ventilation [40].

**Figure 4:**
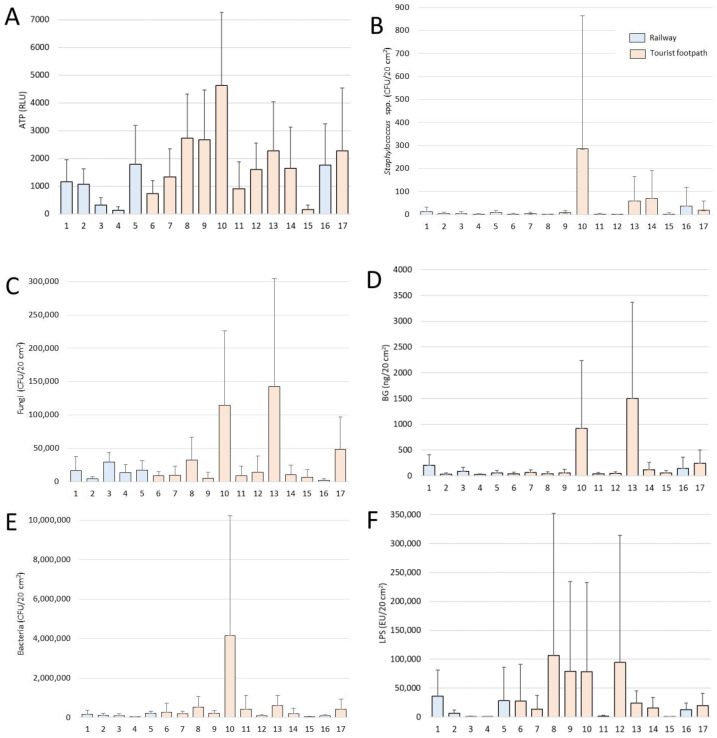
Biomass parameters from experimental periods 2018–2019 and 2019–2020 per individual sites (1–17): (**A**)—ATP as total microbial biomass, (**B**)—*Staphylococcus* spp., (**C**)—fungi, (**D**)—BG, (**E**)—bacteria, (**F**)—LPS. Results are presented as mean ± SD.

LPS and BG are molecules commonly found in pathogens and are elicitors of innate immunity [41]. LPS can cause adverse respiratory symptoms and has a higher acute toxicity than BG. There is strong evidence of links between health problems in workers and exposure to LPS in the workplace [42]. In particular, there is a higher risk in agricultural material processing, food processing, animal husbandry, waste collection, and sanitary waste processing [43]. Although LPS and BG levels might appear to be high at certain sites in the cave (Figure 4, Table S3 in Supplementary Materials), LPS concentrations, for example, are unlikely to exceed occupational exposure limits proposed by several sources: 0.1–0.2 μg/m^3^ [44,45,46,47], 0.025 μg/m^3^ [48], or 0.005 μg/m^3^ [49]. A direct comparison of these values with those from the current study is not possible because of the different experimental setups. To the authors’ knowledge, no comparable study has been conducted so far, especially in karst caves, but it is important to acknowledge the presence of these compounds in cave air. On the other hand, organic molecules, organism fragments, and organisms from the air represent a source of nutrients for the organisms that live in oligotrophic cave environments.

### 4.2. Airborne Staphylococci, Viability and Potential Impact on Humans and Caves

*Staphylococcus* was selected for inclusion in the study as an indicator of human presence [19,20], and to monitor its viability in an otherwise natural environment. *Staphylococcus* spp. are part of the normal human skin microbiota and can also be found on mucous membranes [19,20]. They are commensal microorganisms that play a central role in regulating cutaneous homeostasis and immunocompetence [21]. There are more than 50 different species, and the skin of healthy individuals is colonized by a mixture of these staphylococci, which are present in varying proportions depending upon whether the skin site is dry, moist, or sebaceous [19,21,50].

Survival on different types of surfaces has previously been studied mainly for *Staphylococcus aureus*, the most clinically relevant *Staphylococcus*. These studies have shown that survival is highly dependent upon the type of surface and other environmental conditions, particularly relative humidity and the presence of organic material [51,52]. Interestingly, the survival rate on plastics was higher at lower humidity. The longest observed survival times of *S. aureus* and coagulase-negative staphylococci (CoNS) on non-biological surfaces were 45 days and 51 days, respectively [52,53].

In addition to living in commensal relationship with humans and animals, some *Staphylococcus* species can also cause a variety of mostly pyogenic processes in different parts of the body (e.g., skin, ears, lungs, and joints) [54]. The human nasal microbiota is particularly rich in CoNS. For example, the anterior nostrils of 91.2% of humans are colonized with these bacteria, including most commonly *Staphylococcus epidermidis*, which colonizes 97.1% of the adult population, followed by *S. haemolyticus*, *S. capitis*, *S. hominis*, *S. warneri*, and *S. lugdunensis*, all present within ≥25% of the adult population [50,55,56,57]. The most clinically important species, *S. aureus*, is routinely found in at least 20% of healthy adults [21]. The first, *S. haemolyticus*, is found on the skin of humans and animals (especially cats) and is the second most common cause of community-acquired urinary tract infections in young women, usually after sexual intercourse [54,58]. In addition to cystitis, *S. saprophyticus* can cause foreign body-related infection and native valve endocarditis [55]. The second, *S. equorum*, is associated with fermented foods and the skin of animals (typically horses) [54,55]. It is of low virulence, with no documented clinical cases known to the authors [55]. The third, *S. warneri*, is also found in fermented foods, milk, and on human and animal skin (primarily bovine) [54,55]. It is associated with foreign body-related infections [55]. *S. epidermidis*—widely considered the most common skin isolate—was, however, found only at one sampling site (Table 2, Figure 1).

Only a few of the isolates were assigned to Risk Group 2 (Table 2). These are organisms that can cause disease in humans but are treatable. They usually enter the human body by inhalation of aerosolized cells and spores or by direct contact with contaminated surfaces. These CoNS are emerging as clinically significant pathogens [55]. The most common CoNS, *S. epidermidis*, has been associated with foreign body-related infections, native valve endocarditis, and bloodstream infections [55,57]. Why *S. epidermidis* is of such high clinical significance among CoNS is not yet fully understood. The most likely reason is the high percentage of colonized individuals, which probably increases the likelihood of easy entry of this microbe into the bloodstream and tissues. Other specific virulence factors, other than its great ability to form biofilms on implanted foreign bodies, are not yet known [55,57]. Unlike CoNS, *S. aureus* is an especially important human pathogen that can cause a variety of clinical manifestations, including bacteremia, endocarditis, and osteoarticular, skin, soft tissue, respiratory, and foreign device-related infections. *S. aureus* infections are common, both among the general population and in hospitals, and antibacterial treatment remains a challenge due to the emergence of multidrug-resistant strains [59,60]. During the current study it was detected at only one sampling site (Table 2, Figure 1).

## 5. Conclusions

Natural climatic variations have a remarkable influence upon the distribution of bioaerosol particles. High concentrations of airborne microbial biomass were found at several sites and not only at sites with prolonged human stay. The ATP indicator was best suited for estimating total airborne microbial biomass. Long-term accumulation of bioaerosols was observed at only about one-third of the experimental sites. On inert plastic surfaces, microbial viability decreases over time. Airborne *Staphylococcus* spp. Were found in varying concentrations at all monitoring sites in the cave, but their long-term survival is decreased by high relative humidity, low temperature, the material to which they adhere, and potentially by the radon concentration. The identified staphylococci were commensals and/or opportunistic pathogens. *S. saprophyticus*, *S. equorum*, and *S. warneri* were most prevalent in cave air, and five species (*S. aureus, S. epidermidis, S. haemolyticus, S. pasteuri, S. saprophyticus*) were assigned to Risk Group 2. BG and LPS are present in cave air at widely different concentrations, but their impact on the cave environment and on human visitors has yet to be evaluated.

## Figures and Tables

**Table 1:** Correlation (r) between biomass estimators.

	ATP	BG	LPS	Fungi	Bacteria
ATP	1.000	**0.413**	**0.269**	**0.398**	**0.537**
BG	**0.413**	1.000	–0.010	**0.863**	**0.580**
LPS	**0.269**	–0.010	1.000	0.188	0.044
Fungi	**0.398**	**0.863**	0.188	1.000	**0.588**
Bacteria	**0.537**	**0.580**	0.044	**0.588**	1.000

**in bold**—statistically significant correlation (*p* < 0.05, n = 100).

**Table 2:** *Staphylococcus* isolates (MALDI-TOF MS score value > 2.000, species level identification) identified in Postojnska jama.

Species	Site	Risk Group (Country)
*S. arlettae*	1, 13	–
*S. aureus*	16	2 (US, NZ, BE, CA, EU, CH, UK)
*S. chonii*	13, 14	–
*S. epidermidis*	4	2 (BE, DE, CH)
*S. equorum*	5, 10, 13, 14, 15	–
*S. equorum ssp. equorum*	13	–
*S. haemolyticus*	10	2 (DE, CH)
*S. pasteuri*	10	2 (DE, CH)
*S. saprophyticus*	1, 3, 4, 6, 7, 13, 14, 15, 17	2 (BE, CA, DE, CH)
*S. sciuri*	6, 11	–
*S. warneri*	2, 5, 10, 12	–
*S. xylosus*	1	–

BE—Belgium; CA—Canada; CH—Switzerland; DE—Germany; EU—European Union; NZ—New Zealand; UK—United Kingdom; US—United States of America.

## Data Availability

Data is contained within the article or supplementary material.

## Supplementary Materials

The following supporting information can be downloaded at: https://www.mdpi.com/article/10.3390/microorganisms11030809/s1, Table S1: Average values of climatic parameters from June 2020 to June 2021, and concentration of settled airborne biomass with CV (expressed in RLU of ATP) from April to November 2020; Table S2: Biomass dynamics during bioaerosol settling experiments between January 2018–January 2019 (aerosols analysed after 3, 6, and 12 months) and January 2019–November 2020 (aerosols analysed after 7, 13, and 22 months); Table S3: Ranges of measured biomass parameters (2018–2019 and 2019–2020).
