# Supplementary material for: Long-Term Monitoring of Bioaerosols in an Environment without UV and Desiccation Stress, an Example from the Cave Postojnska Jama, Slovenia
Source: Microorganisms. 2023 Mar 22;11(3):809. doi: 10.3390/microorganisms11030809 (PMC10053050; doi:10.3390/microorganisms11030809)
Supplement: Supplementary file 1 [file microorganisms-11-00809-s001.zip › microorganisms-2273278-supplementary.pdf]

Table S1. Average values of climatic parameters from June 2020 to June 2021, and concentration of settled airborne biomass with CV (expressed in RLU of ATP) from April to November 2020.

Site	Main impact	Air velocity (m/s)	Air flow (l/s)	Air temperature (°C)	ATP ± SD (RLU)	CV (%)
1	River, train	0.05 ± 0.06	3.77 ± 4.35	**11.0 ± 3.9**	3672.7 ± 1324.7	36.1
2	Train	0.08 ± 0.12	6.20 ± 8.69	9.0 ± 1.3	1376.5 ± 846.9	61.5
3	Train	0.02 ± 0.04	1.65 ± 2.77	9.2 ± 0.5	542.7 ± 218.4	40.3
4	Train	0.01 ± 0.03	0.87 ± 1.82	9.4 ± 0.3	182.3 ± 30.9	16.9
5	Train	0.07 ± 0.08	4.84 ± 5.52	9.5 ± 0.3	1406.3 ± 268.6	19.1
6	Visitors	0.01 ± 0.01	0.56 ± 0.53	10.3 ± 0.2	253.5 ± 167.8	66.2
7	Visitors	0.01 ± 0.01	0.79 ± 0.62	10.3 ± 0.2	623.0 ± 220.0	35.3
8	Visitors	0.01 ± 0.02	1.05 ± 1.67	10.3 ± 0.2	463.7 ± 37.0	8.0
9	Visitors	0.01 ± 0.02	1.00 ± 1.18	10.2 ± 0.2	1520.3 ± 1573.2	103.5
10	Visitors	0.01 ± 0.01	0.46 ± 0.65	10.1 ± 0.2	2286.7 ± 3239.8	141.7
11	Visitors	0.01 ± 0.02	0.72 ± 1.18	10.1 ± 0.2	**4780.7 ± 2981.1**	62.4
12	Visitors*	0.02 ± 0.04	1.75 ± 3.04	9.6 ± 0.1	347.3 ± 125.8	36.2
13	Visitors	0.02 ± 0.03	1.44 ± 1.85	9.7 ± 0.1	1695.3 ± 1840.1	108.5
14	Visitors	0.00 ± 0.01	0.32 ± 0.23	9.8 ± 0.1	1074.5 ± 2352.5	**218.9**
15	Visitors	0.02 ± 0.02	1.31 ± 1.43	10.3 ± 0.2	655.3 ± 158.5	24.2
16	Train	**0.11 ± 0.11**	**7.82 ± 8.20**	10.4 ± 0.3	687.8 ± 450.6	65.5
17	Visitors	0.01 ± 0.01	0.54 ± 0.64	11.0 ± 0.5	2593.7 ± 725.3	28.0
1–17		0.04 ± 0.08	2.65 ± 5.72	10.1 ± 1.6	1479.4 ± 1703.2	115.1

in bold – site with the highest value of the parameter; * – site located 20 m above the tourist footpath

Table S2. Biomass dynamics during bioaerosol settling experiments between January 2018–January 2019 (aerosols analysed after 3, 6, and 12 months) and January 2019–November 2020 (aerosols analysed after 7, 13, and 22 months)

Site	Experiment (Years: Months)	Fungi	Bacteria	*Staphylococcus*	ATP	LPS	BG
1	2018–2019: 3–6–12	–	–	0	+	±	+
	2019–2020: 7–13–22	±	+	–	+	±	–
2	2018–2019: 3–6–12	+	±	0	±	–	+
	2019–2020: 7–13–22	±	+	±	±	±	±
3	2018–2019: 3–6–12	±	±	±	±	±	±
	2019–2020: 7–13–22	±	±	±	±	±	±
4	2018–2019: 3–6–12	+	+	±	+	+	±
	2019–2020: 7–13–22	+	+	+	–	±	±
5	2018–2019: 3–6–12	+	–	±	±	±	+
	2019–2020: 7–13–22	±	±	–	±	+	±
6	2018–2019: 3–6–12	+	±	0	±	±	+
	2019–2020: 7–13–22	±	±	–	+	±	±
7	2018–2019: 3–6–12	+	±	±	+	±	+
	2019–2020: 7–13–22	±	±	±	±	+	+
8	2018–2019: 3–6–12	±	±	0	±	±	+
	2019–2020: 7–13–22	±	±	±	–	±	+
9	2018–2019: 3–6–12	±	±	0	±	±	±
	2019–2020: 7–13–22	±	–	±	–	+	+
10	2018–2019: 3–6–12	±	+	±	+	±	+
	2019–2020: 7–13–22	±	±	–	±	±	+
11	2018–2019: 3–6–12	+	+	±	+	+	+
	2019–2020: 7–13–22	+	+	+	+	±	±
12	2018–2019: 3–6–12	±	±	0	+	±	±
	2019–2020: 7–13–22	±	±	±	–	±	±
13	2018–2019: 3–6–12	+	+	±	+	+	+
	2019–2020: 7–13–22	±	+	–	+	+	+
14	2018–2019: 3–6–12	+	+	±	±	±	+
	2019–2020: 7–13–22	±	±	–	±	–	±
15	2018–2019: 3–6–12	+	+	0	+	+	+
	2019–2020: 7–13–22	±	±	±	±	+	±
16	2018–2019: 3–6–12	±	±	0	+	+	+
	2019–2020: 7–13–22	±	±	–	–	±	±
17	2018–2019: 3–6–12	±	±	±	+	±	+
	2019–2020: 7–13–22	±	+	±	±	±	±
	**Accumulation (%)**	32.4	35.3	5.9	41.2	29.4	52.9
	**Reduction (%)**	2.9	8.8	20.6	14.7	5.9	2.9
	**Equivocal (%)**	64.7	55.9	50.0	44.1	64.7	44.1
	**"0" (%)**	0.0	0.0	23.5			

"+" – accumulation; "–" – reduction; "±" – equivocal; "0" – 0 CFU/20 cm^2

Table S3. Ranges of measured biomass parameters (2018–2019 and 2019–2020)

Site	ATP (RLU)	Fungi (CFU/20 cm^2)	Bacteria (CFU/20 cm^2)	*Staphylococcus* (CFU/20 cm^2)	LPS (EU/20 cm^2)	BG (ng/20 cm^2)
1	539–2,691	2,515–55,645	27,037–514,640	0–50	1,258–113,901	17–555
2	508–2,064	635–10,689	24,522–285,454	0–16	1,182–17,527	3–66
3	45–748	13,518–52,816	9,117–268,481	0–19	138–1,547	8–187
4	1–374	289–32,067	113–59,984	0–6	2–503	1–52
5	220–3,456	1,050–43,384	33,953–319,410	0–22	843–145,068	13–114
6	14–1,286	270–18,234	104–1.186,261	0–11	20–157,190	2–85
7	59–2,842	0–33,953	65,416–414,982	0–14	250–61,241	11–144
8	391–4,613	553–92,294	145,872–1.580,422	0–3	**2,339–608,376**	1–86
9	422–4,635	817–21,315	66,020–362,795	0–20	3,408–356,671	5–175
10	**944–7,318**	8,252–290,487	**138,972–13.518,346**	**0–1462**	5,890–393,780	2–2.796
11	22–2,471	176–36,782	1,091–1.765,433	0–10	33–4,112	5–76
12	285–2,700	0–62,876	15,071–180,976	0–3	966–541,991	11–92
13	20–4,391	**651–420,012**	73,565–1.273,240	0–264	478–48,377	**13–4.729**
14	3–4,255	44–37,726	119–773,375	0–310	39–49,320	1–360
15	1–405	57–27,980	233–61,619	0–14	2–1,346	3–120
16	87–3,768	0–6,033	11,091–192,420	0–183	4,616–32,184	3–524
17	50–5,290	3207–115,482	54,193–1.383,273	0–102	6,275–60,991	2–589

In bold – site with the highest value of the parameter.
